# Editorial: Current perspectives on the use of entomopathogenic fungi for pest control

**DOI:** 10.3389/ffunb.2025.1609506

**Published:** 2025-05-07

**Authors:** Isabele Costa Angelo, José Luis Ramírez, Adalberto Á. Pérez de León, Patricia Silva Gôlo

**Affiliations:** ^1^ Department of Epidemiology and Public Health, Veterinary Institute, Federal Rural University of Rio de Janeiro, Seropédica, RJ, Brazil; ^2^ Crop Bioprotection Research Unit, National Center for Agricultural Utilization Research, United States Department of Agriculture – Agricultural Research Service (USDA-ARS), Peoria, IL, United States; ^3^ San Joaquin Valley Agricultural Sciences Center, United States Department of Agriculture – Agricultural Research Service (USDA-ARS), Parlier, CA, United States; ^4^ Department of Animal Parasitology, Veterinary Institute, Federal Rural University of Rio de Janeiro, Seropédica, RJ, Brazil

**Keywords:** biological control, mycopesticide formulation, characterization of entomopathogenic fungi, *Metarhizium* genomics, *Beauveria* sp., sustainability, biopesticide

**Figure 1:**
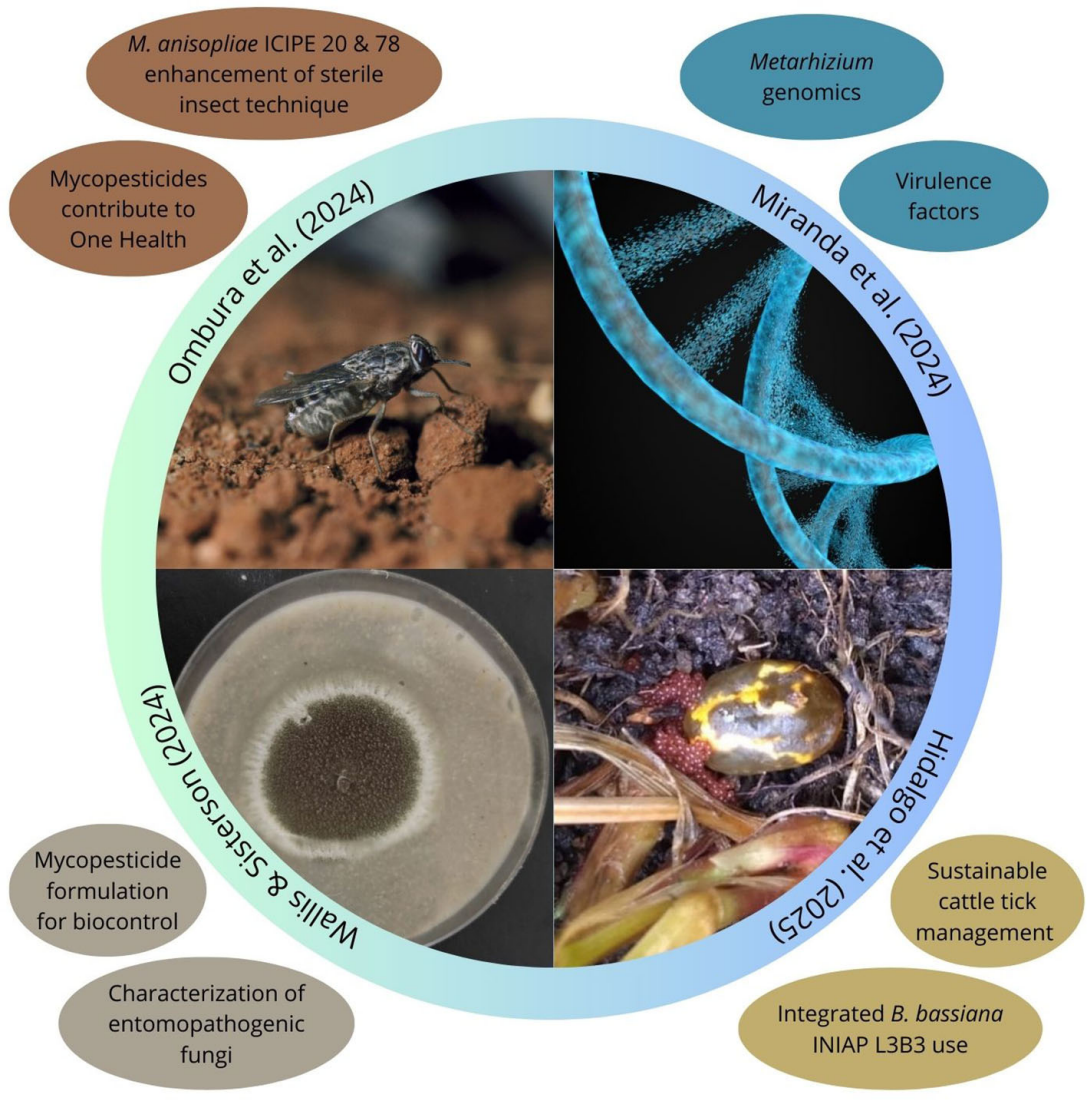
Illustration of editorial for publications in the Research Topic, “Current perspectives on the use of entomopathogenic fungi for pest control”. Quadrants synthesize topics covered in each publication. This figure was assembled using elements from Canva.com.

Scientific and technological advancements since 1835 when Dr. Agostino Bassi discovered the entomopathogenic fungus now known as *Beauveria bassiana* continue to expand the research and development of mycopesticides as sustainable alternatives to synthetic chemical pesticides for arthropod pest management in agricultural, veterinary, and public health settings (Bassi, 1836; Angelo et al., 2022). Progress in the formulation of mycopesticides allows their integrated use with other management technologies to mitigate overreliance on synthetic pesticides and the emergence of pest resistant populations, which contributes to human and environmental health (Bamisile et al., 2021; Mascarin et al., 2024). This Research Topic encompasses 4 studies (**Figure 1**) that highlight novel applications of entomopathogenic fungi, from optimizing fungal formulations and expanding the diversity of applicable strains to integrating innovative techniques for arthropod pest and vector control with broad agricultural and public health implications. The contributions of Wallis and Sisterson, Miranda et al., Ombura et al., and Hidalgo et al. synthesized in this editorial showcase significant progress with, and underscore key challenges on the use of entomopathogenic fungi for arthropod pest control.

Wallis and Sisterson examined the use of fungal-based agents for pest management in orchards and vineyards stressing the importance of expanding the diversity of available strains by standardizing screening and testing methods, which could lead to the discovery of more virulent and resilient isolates. Another critical point is the development of innovative formulations, such as microencapsulation, the use of UV protectants, and slow-release carriers, to enhance fungal environmental persistence. Notably, a multidisciplinary approach that integrates biotechnology, microbial ecology, and agricultural sciences is needed to transition mycopesticidal formulations from the laboratory to large-scale field applications.

Miranda et al. complemented this perspective providing a comprehensive review of host-pathogen interactions with *Metarhizium* species that details the mechanisms of cuticle penetration and the production of virulence factors demonstrating their potential for controlling a wide range of insect pests and disease vectors. Recent advances in genomics and genetic engineering enabled the development of strains with enhanced specificity and environmental tolerance. This review bridged molecular insights with practical biocontrol strategies and linked advances in genomics, transcriptomics, and genetic manipulation to real-world applications.

Ombura et al. explored an innovative strategy for controlling the tsetse fly, which is the vector of pathogens causing African trypanosomiasis, that combines sterile insect technique (SIT) with fungal infection using *M*. *anisopliae* strains ICIPE 20 and 78. Sterilized male flies inoculated with fungal conidia enabled horizontal transmission to females and other males during mating or social interactions. This dual approach could significantly reduce the vector population via sterility and the intensified pesticidal effect through horizontal transmission of entomopathogenic fungus. If validated in the field, this approach would be a cost-effective and environmentally-friendly alternative to chemical trypanocides thus contributing to One Health objectives by reducing the reliance on synthetic chemical pesticides and mitigating the development of antimicrobial resistance (Percoma et al., 2018; Kasozi et al., 2024).

Hidalgo et al. summarized efforts in Ecuador addressing the growing challenge of acaricide-resistant populations of the one-host tick *Rhipicephalus microplus*, an economically important ectoparasite and disease vector affecting cattle. Their work focused on the development of a mycopesticide using *B*. *bassiana* sensu lato strain INIAP L3B3. A phased approach going from strain selection and efficacy testing to formulation optimization and safety assessment demonstrated the acaricidal potential of INIAP L3B3 against various life stages of *R*. *microplus*. Pilot field trials indicated comparable efficacy to conventional chemical acaricides, and additional studies showed synergistic effects when *B*. *bassiana* INIAP L3B3 was combined with certain botanical extracts and synthetic acaricides. Advances in conidial production and formulation that will support its practical application and commercialization were documented. These findings underscore the viability of *B*. *bassiana* INIAP L3B3 as a component of integrated tick management strategies but also provides a model for other entomopathogenic fungi as part of sustainable pest control solutions in livestock systems.

Collectively, the 4 studies revealed a promising scenario for the use of entomopathogenic fungi as tools for integrated pest and disease vector management. Each work presented a unique perspective, from optimizing biological agents and expanding the strain repertoire to implementing combined strategies and validating sustainable alternatives across agriculture, public health, and livestock production. This diversity of approaches underscores the importance of investing in research that spans from molecular mechanisms and genomic insights to practical applications and product formulation designed for real-world environments.

The need to develop more robust entomopathogenic fungal strains capable of withstanding climatic variations and adapting to different environmental conditions is a common theme identified by Wallis and Sisterson and reinforced by the other studies. Biotechnological enhancement through the integration of innovative techniques, such as combining SIT with fungal infection as proposed by Ombura et al., opens new perspectives for the sustainable suppression of vector populations without the adverse effects associated with intense chemical use. Meanwhile, the reviews by Miranda et al. and Hidalgo et al. demonstrated that strains of entomopathogenic fungi like *Metarhizium* and *Beauveria* have comparable efficacy to conventional control methods, and under certain circumstances could potentially replace or complement synthetic insecticides and acaricides, thereby contributing to the sustainability of production systems and mitigation of environmental and health risks. Collectively, these studies also emphasize the need for interdisciplinary research that incorporates biotechnology, microbial ecology, genetic engineering, and agronomic management to overcome the inherent challenges in the development and commercialization of mycopesticides.

Studies in this Research Topic demonstrated the central role entomopathogenic fungi can play in sustainable pest control. The diverse approaches and innovative solutions discussed emphasize how basic and applied research can deliver robust solutions to the challenges of pest and disease vector management. Interdisciplinary and collaborative efforts among universities, research institutions, and industry are essential to develop effective formulations of entomopathogenic fungi that are used globally for safer pest control. This will fully realize the contribution of mycopesticides to food security, public health, and animal welfare.
